# Supplementary material for: Apoptotic tumor cell-derived microRNA-375 uses CD36 to alter the tumor-associated macrophage phenotype
Source: Nat Commun. 2019 Mar 8;10:1135. doi: 10.1038/s41467-019-08989-2 (PMC6408494; doi:10.1038/s41467-019-08989-2)
Supplement: Supplementary file 1 — Supplementary Information [file 41467_2019_8989_MOESM1_ESM.pdf]

Supplementary information for:

Apoptotic tumor cell-derived microRNA-375 uses CD36 to alter the tumor-associated macrophage phenotype

Frank *et al.*

Description of Additional Supplementary Files

File Name: Supplementary Data 1
Differentially expressed miRNA genes in miRseq of primary human MΦ control, cocultured with MCF-7 cells, and treated with RvD1. Normalized mature miRNA expression table using multi mapper strategy by number of reads per million (RPM).

File Name: Supplementary Data 2
Potential direct miR-375 targets identified in primary human MΦ by Ago-RIP-Seq. Normalized mRNA expression table of linear number of reads per kilobase of transcripts per million mapped reads (RPKM).

File Name: Supplementary Table 1
List of all human and mouse qPCR primers used in this study, including their sequences.

File Name: Supplementary Table 2
List of all cell lines used in this study along with their supplier and their specific culture conditions.

File Name: Supplementary Movie 1
3D volume rendering of control MCF-7 spheroids infiltrated with eFluor670 labeled CD14+ monocytes. Area occupied by infiltrating monocytes is denoted by white, whereas total spheroid area is in blue.

File Name: Supplementary Movie 2
3D volume rendering of miR-375 decoy MCF-7 spheroids infiltrated with eFluor670 labeled CD14+ monocytes. Area occupied by infiltrating monocytes is denoted by white, whereas total spheroid area is in blue.

Supplementary figures

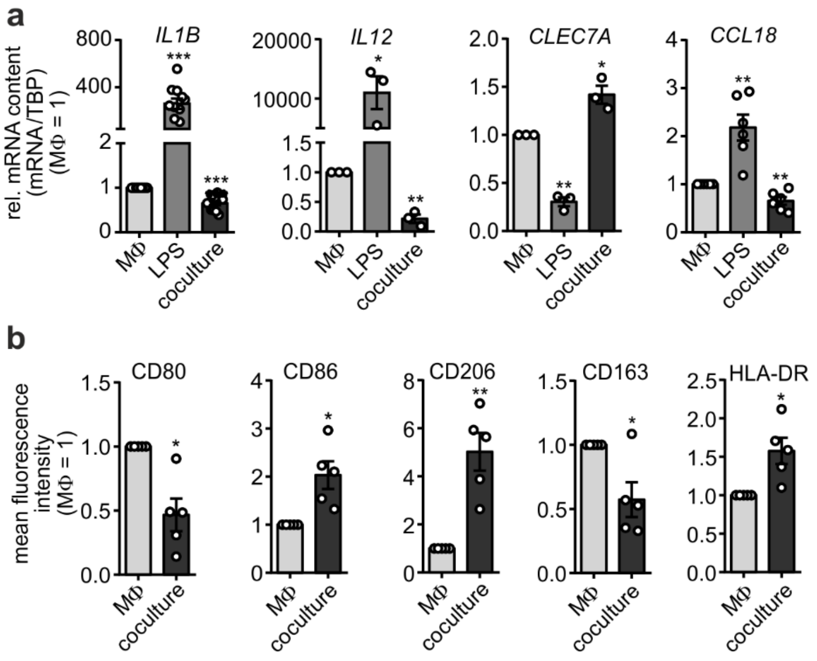

Supplementary Fig. 1 - MCF-7 coculture with human primary MΦ reprograms them to TAMs. (**a** and **b**) Primary human monocyte-derived MΦ were cocultured with MCF-7 cells for 48 h (coculture). Residual MCF-7 cells were removed and MΦ were harvested. **a** In parallel, naïve MΦ were treated with 1 µg/mL LPS for 6 h (LPS). *IL1B*, *IL12*, *CLEC7A* and *CCL18* mRNA expressions were analyzed by qPCR and normalized to control MΦ (MΦ). Data are mean ± SEM of n ≥ 3. **b** MΦ were analyzed by polychromatic flow cytometry for CD80, CD86, CD206, CD163, and HLA-DR. Mean fluorescence intensities were normalized to control MΦ. Data are mean ± SEM of n = 5. P-values were calculated using one-sample t test. *, $p < 0.05$, **, $p < 0.01$, ***, $p < 0.001$.

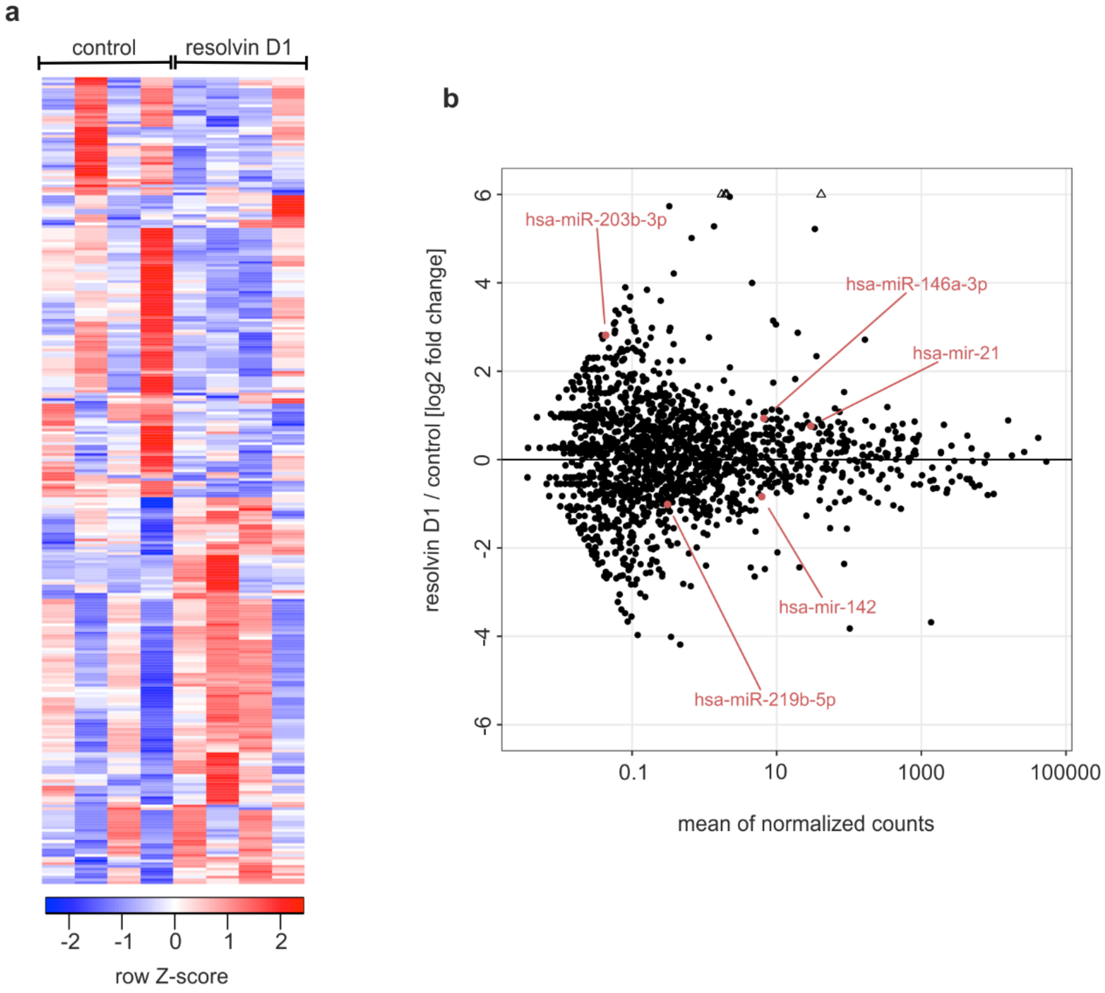

Supplementary Fig. 2 - MiR signature in human MΦ treated with resolvin D1. **a** A heatmap of differentially expressed miRs from control and human MΦ treated with 100 nM resolvin D1 for 6 h is shown (n = 4 each). **b** *MA*-plot (log ratio mean average) of differentially expressed miRs in control and treated MΦ. Highly expressed miR-21, miR-146, miR-142, miR-203 and miR-219 (PMID: 24692596, 22957142) are denoted in red.

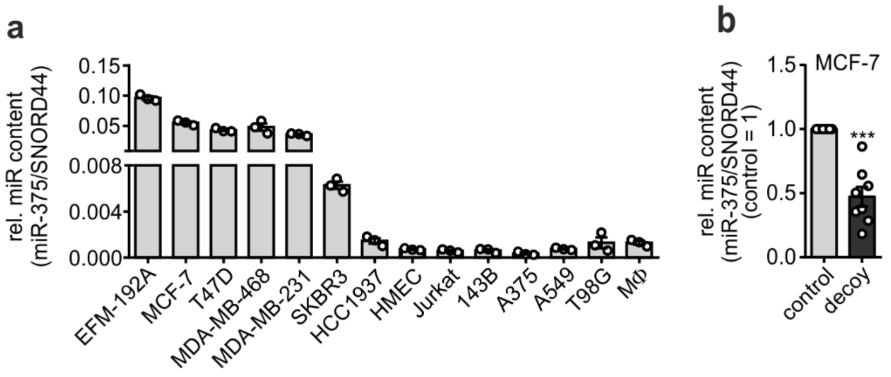

Supplementary Fig. 3 - Breast cancer cell lines have a high constitutive miR-375 expression. **a** Expression of miR-375 was measured via qPCR in different ER+ (EFM-192A, MCF-7, T47D) and ER- (MDA-MB-468, MDA-MB-231, SKBR3, HCC1937) breast carcinoma cells, primary mammary epithelial cells (HMEC), Jurkat T cell leukemia, 143B osteosarcoma, A375 malignant melanoma, A549 lung carcinoma, T98G glioblastoma as well as primary human MΦ. Data are mean ± SEM of n = 3. **b** Lentiviral mediated stable knockdown of miR-375 in MCF-7 cells using a miR-375 decoy (decoy) or empty vector (control). miR-375 expression was quantified by qPCR and is shown relative to MCF-7 control. Data are mean ± SEM of n = 8. P-values were calculated using one-sample t test. ***, $p < 0.001$.

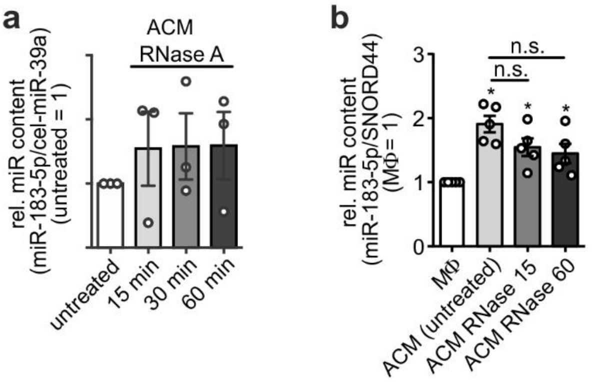

Supplementary Fig. 4 - MiR-183-5p abundance in MCF-7 cell ACM after RNase A treatment. **a** ACM was incubated with 50 µg/ml RNase A at 37°C for times indicated. As a control, ACM was incubated at 37°C for 60 min without RNase A. Prior to RNA isolation, cel-miR-39a was added as a spike-in and normalization control. miR-183-5p abundance was quantified via qPCR and normalized to control ACM. Data are mean ± SEM of *n* = 2-3. **b** MΦ were incubated for 30 min with MCF-7 control ACM or ACM that was treated with 50 µg/ml RNase A for 15 or 60 min. Cells were washed and cultured for another 24 h in MΦ media. miR-183-5p abundance was determined via qPCR and normalized to untreated MΦ control (MΦ = 1). Data are mean ± SEM of *n* = 5. P-values were calculated using one-sample *t* test. *, $p < 0.05$; n.s.: not significant.

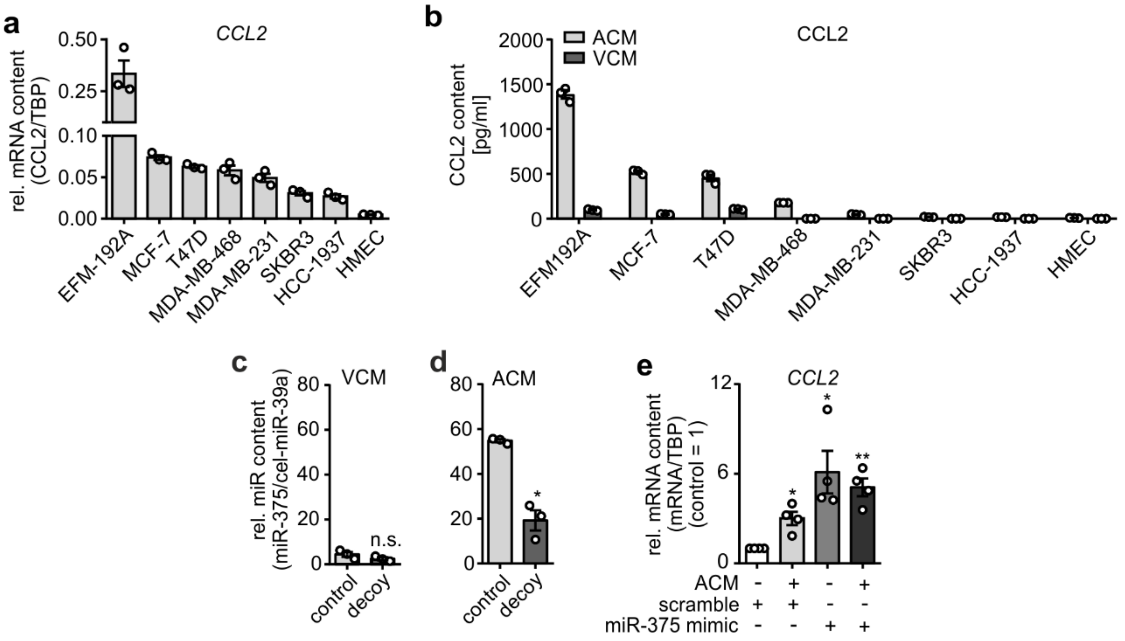

Supplementary Fig. 5 - a *CCL2* mRNA expression of different breast cancer cell lines (ER+ cells; EFM-192A, MCF-7, T47D and ER- cells; MDA-MB-468, MDA-MB-231, SKBR3, HCC1937) and primary human mammary epithelial cells (HMEC) was measured by qPCR. **b** VCM and ACM of different breast cancer cell lines (ER+ cells; EFM-192A, MCF-7, T47D and ER- cells; MDA-MB-468, MDA-MB-231, SKBR3, HCC1937) and primary human mammary epithelial cells (HMEC) were analyzed for CCL2 protein expression by cytometric bead array. Data are mean ± SEM of $n = 3$. (**c** and **d**) Control empty vector (control) MCF-7 or miR-375 decoy (decoy) MCF-7 VCM/ACM was analyzed for the abundance of miR-375 by qPCR. Data are mean ± SEM of $n = 3$. P-values were calculated using one-sample *t* test. *, $p < 0.05$; n.s.: not significant. **e** Primary human MΦ were transfected with synthetic miR-375 mimic or negative control cel-miR-39a (scramble) for 24 h followed by treatment with MCF-7 cell ACM for 30 min. Cells were washed and fresh MΦ media was added for 24 h. *CCL2* mRNA expressions in MΦ were measured by qPCR and normalized to scramble transfected MΦ. Data are mean ± SEM of $n = 4$. P-values of were calculated using one-sample *t* test *, $p < 0.05$, **, $p < 0.01$.

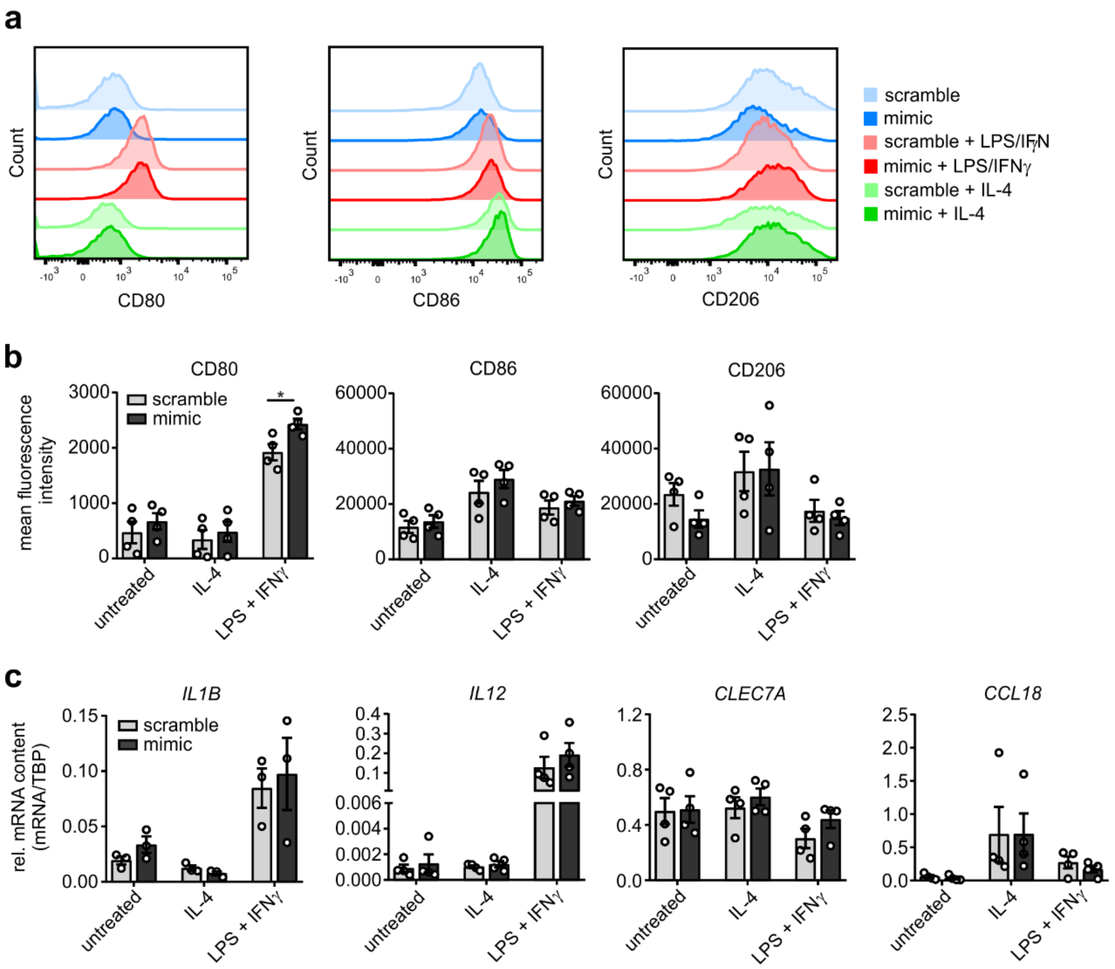

Supplemental Fig. 6 - Role of miR-375 in MΦ polarization. (**a** - **c**) Primary human monocyte-derived MΦ were transfected with synthetic miR-375 mimic or control transfected with cel-miR-39a (scramble). Both, control and miR-375 overexpressing MΦ were then treated with 20 ng/mL IL-4 for 48 h or 100 ng/mL LPS together with 100 U/mL IFNγ for 24 h, respectively. After a total of 72 h after transfection MΦ were harvested and **a** and **b** analyzed by polychromatic flow cytometry for CD80, CD86 and CD206 and **c** mRNA expression of *IL1B*, *IL12*, *CLEC7A* and *CCL18* was analyzed via qPCR. Data are mean ± SEM of n ≥ 3.

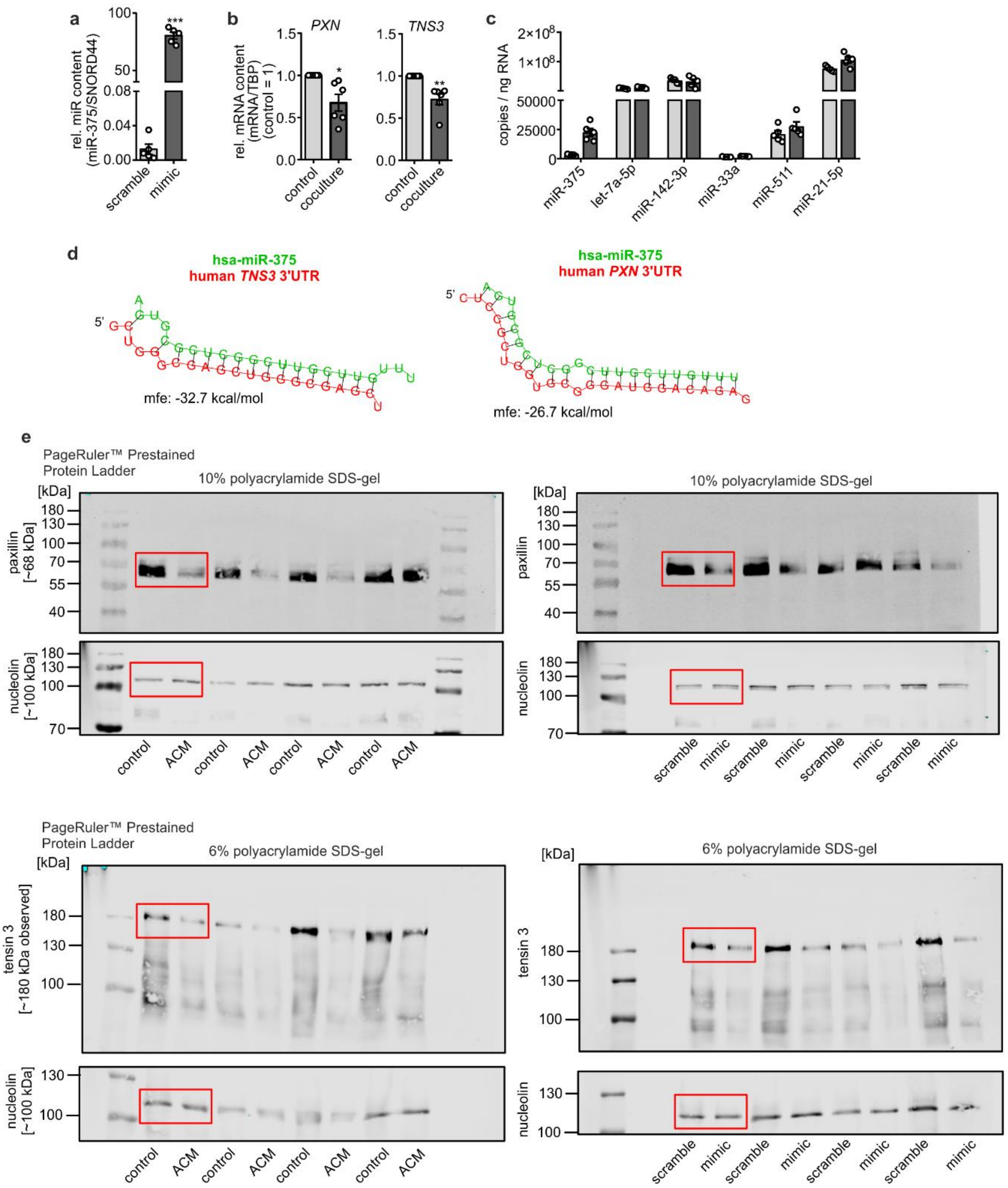

Supplementary Fig. 7 - a Overexpression of miR-375 in primary human monocyte-derived MΦ. Primary human monocyte-derived MΦ were transfected with synthetic miR-375 mimic or transfected with control cel-miR-39a (scramble) for 72 h. The expression of miR-375 was analyzed via qPCR. Data are mean ± SEM of n = 5. **b** MΦ were cocultured with MCF-7 cells for 72 h. MΦ were harvested and analyzed for mRNA expressions of *PXN* and *TNS3* by qPCR. Data are normalized to untreated control MΦ and mean ± SEM of n = 6 are shown. P-values

were calculated using one-sample *t* test. *, $p < 0.05$, **, $p < 0.01$, ***, $p < 0.001$. **c** miR copy numbers in human primary macrophages. MΦ were treated with ACM of MCF-7 cells for 30 min (ACM) or left untreated (control). Cells were washed, fresh media was added for 24 h, cells were harvested and the number of copies per ng total RNA was calculated for individual miRs. Data are mean ± SEM of $n = 6$. **d** uncropped blots used in Fig. 5a, b). **e** Analysis of 3'UTR of human tensin 3 and paxillin for miR-375 binding with the RNAhybrid tool (PMID: 15383676). Negative minimum free energy (mfe) signifies physiological binding.

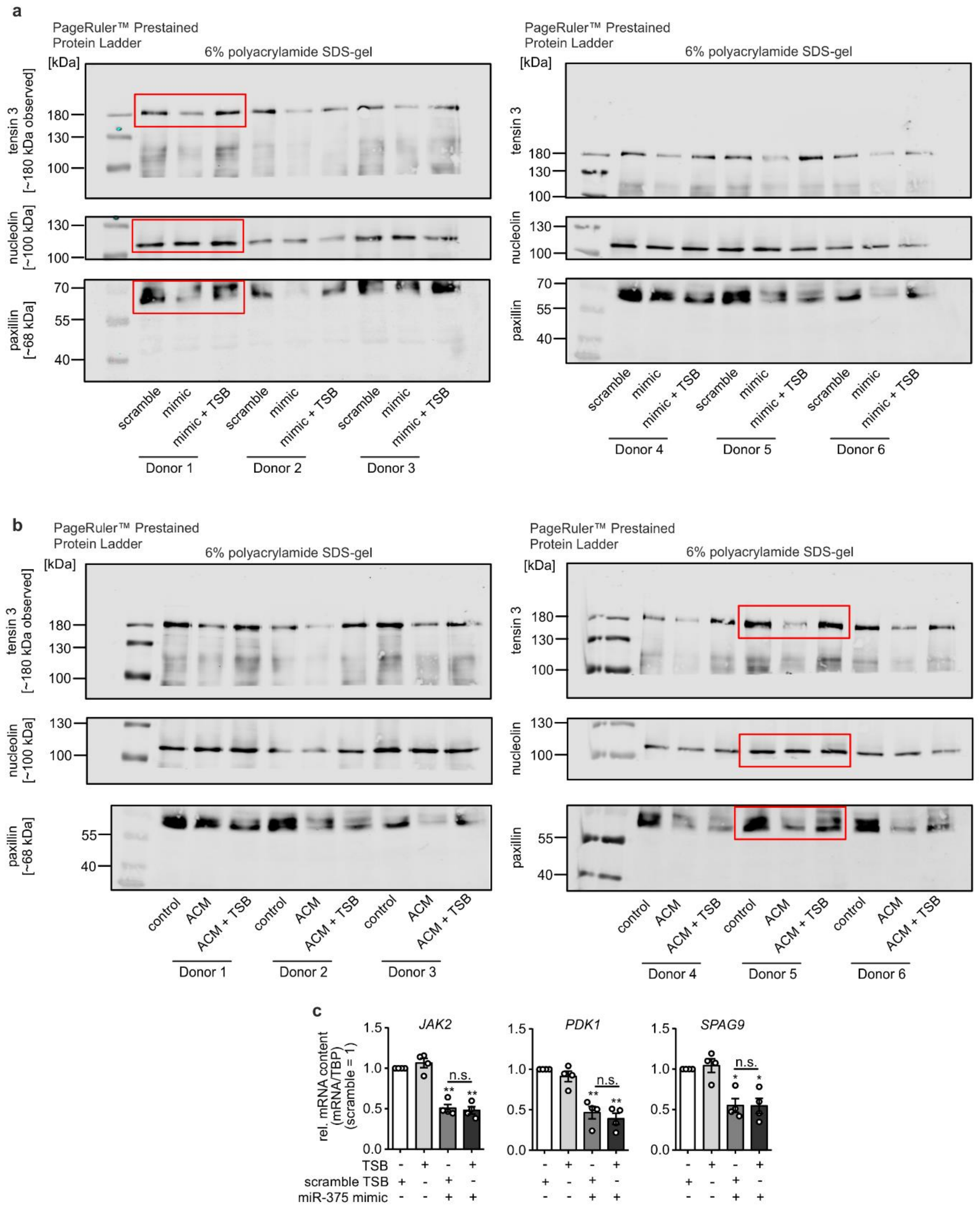

Supplementary Fig. 8 - miR-375 target site blockers for PXN and TNS3 activity. **a** Uncropped blots used in Fig. 6c. **b** Uncropped blots used in Fig. 6e. **c** MΦ were transfected with synthetic miR-375 mimic in the presence of scramble TSBs or *PXN* and *TNS3* specific miR-375 TSBs for 72 h. mRNA expression of miR-375 target genes *JAK2*, *PDK1* and *SPAG9* were measured

in MΦ by qPCR. Data are mean ± SEM of $n = 4$. P-values were calculated using two-tailed Student's *t*-test. *, $p < 0.05$, **, $p < 0.01$, n.s.: not significant.

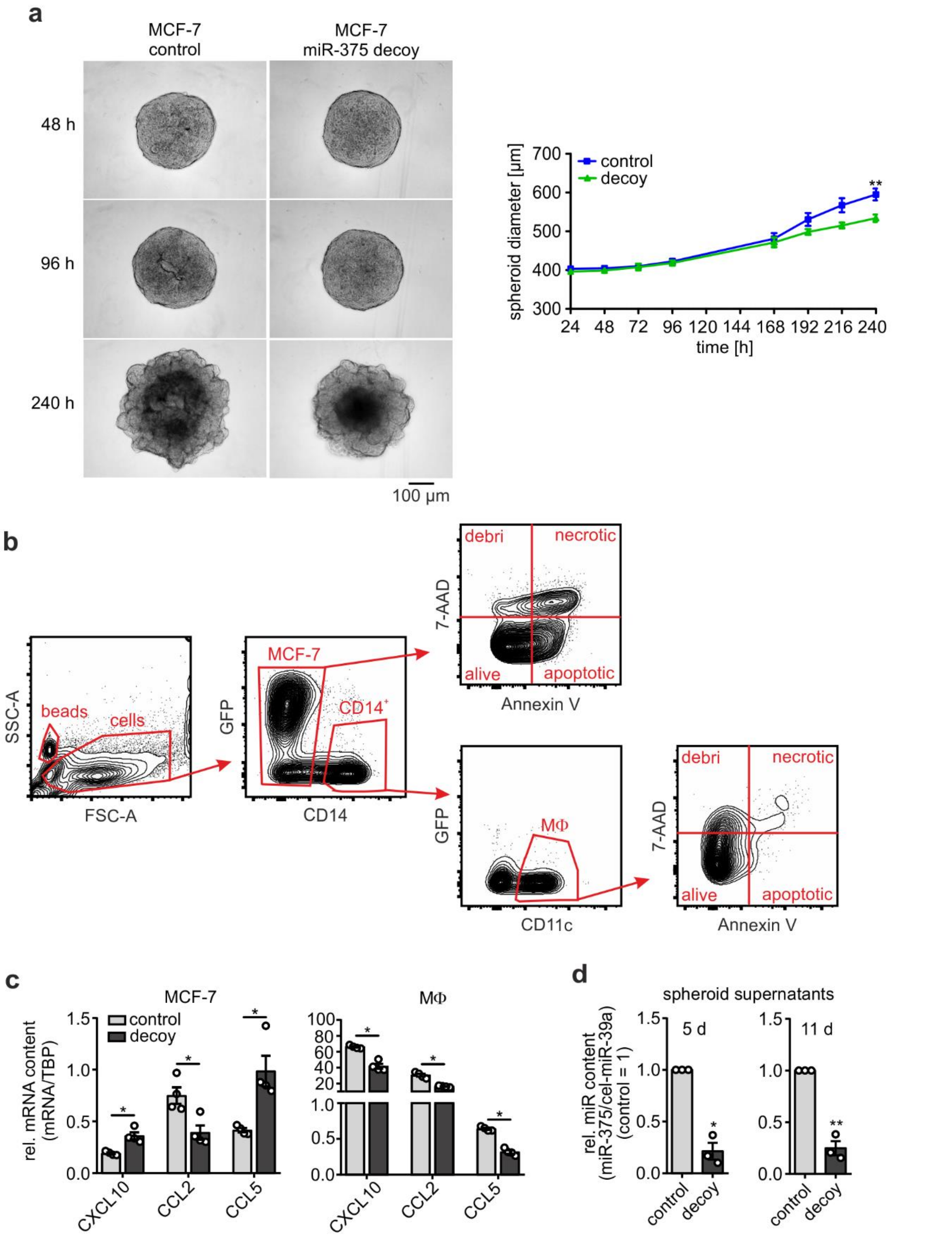

Supplementary Fig. 9 - MCF-7 control and decoy spheroids were generated from 5 x 10^3 MCF-7 cells by the liquid-overlay technique. **a** Tumor growth of MCF-7 spheroids was monitored for 240 h. Pictures were taken at times indicated and spheroid diameters were measured. Data are mean ± SEM of *n* = 3 and 5 individual spheroids for each group. P-values

were calculated using nonparametric two-tailed Student's *t* test **, $p < 0.01$. (**b** - **d**) Spheroids were infiltrated with 5 x 10^5 $CD14^+$ monocytes for three days. **b** Gating strategy to discriminate MCF-7 cells and MΦ via flow cytometry. As an internal counting standard for later analysis, flow-count fluorospheres (beads) were gated. Single-cell populations were gated for GFP-expressing MCF-7 control or decoy cells and $CD14^+$ cells, which were further gated for $CD11c^+$ MΦ. Both, MCF-7 cells and $CD14^+$ MΦ were then analyzed for the amount of apoptosis and necrosis by 7-AAD and Annexin V staining. **c** Spheroid infiltrating MΦ were separated from MCF-7 cells via CD14 microbeads followed by analysis of chemokine mRNA expression in both cell fractions by qPCR. Data are normalized to MCF-7 controls and represent the mean ± SEM of $n = 4$. P-value was calculated using two-tailed Student's *t* test; *, $p < 0.05$. **d** Spheroid supernatants were harvested at indicated times and analyzed for the abundance of miR-375 by qPCR. Data are normalized to control MCF-7 and represents mean ± SEM of $n = 3$. P-values were calculated using one-sample t test. *, $p < 0.05$, **, $p < 0.01$.

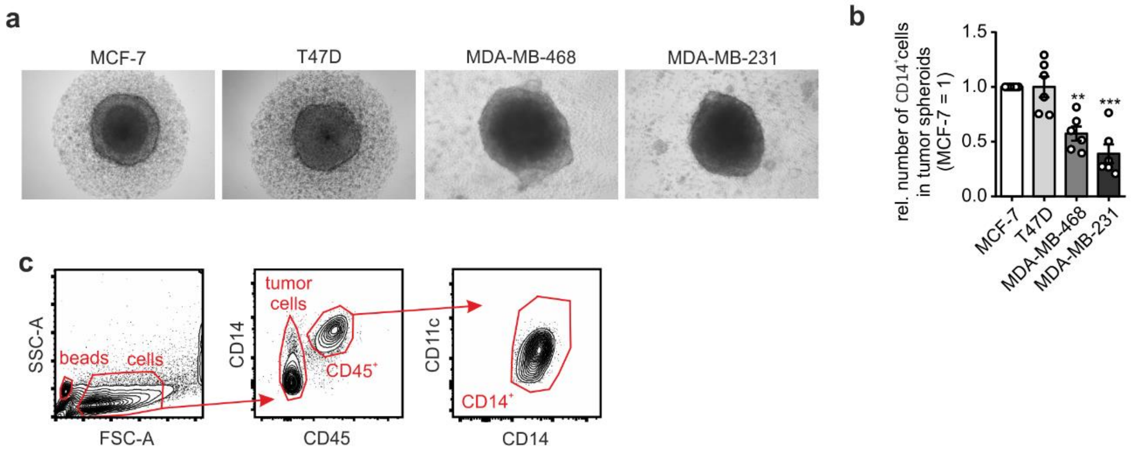

Supplementary Fig. 10 - Infiltration of CD14$^+$ cell in different ER$^+$ (MCF-7, T47D) and ER$^-$ (MDA-MB-468, MDA-MB-231) spheroids. **a** Representative pictures of tumor-spheroid CD14$^+$ cell cocultures after three days of infiltration. **b** Cocultures were harvested and non-infiltrating cells were removed. Single-cell suspensions of infiltrated spheroids were analyzed via polychromatic flow cytometry for the number of MΦ in tumor spheroids. Data are mean ± SEM of n = 6. P-values were calculated using two-tailed Student's t-test. **, $p < 0.01$, ***, $p < 0.001$. **c** Gating strategy to discriminate tumor cells and infiltrated MΦ by flow cytometry. Single-cell suspensions were gated for CD14$^+$/CD45$^+$ immune cells followed by gating for CD11c$^+$/CD14$^+$ MΦ.

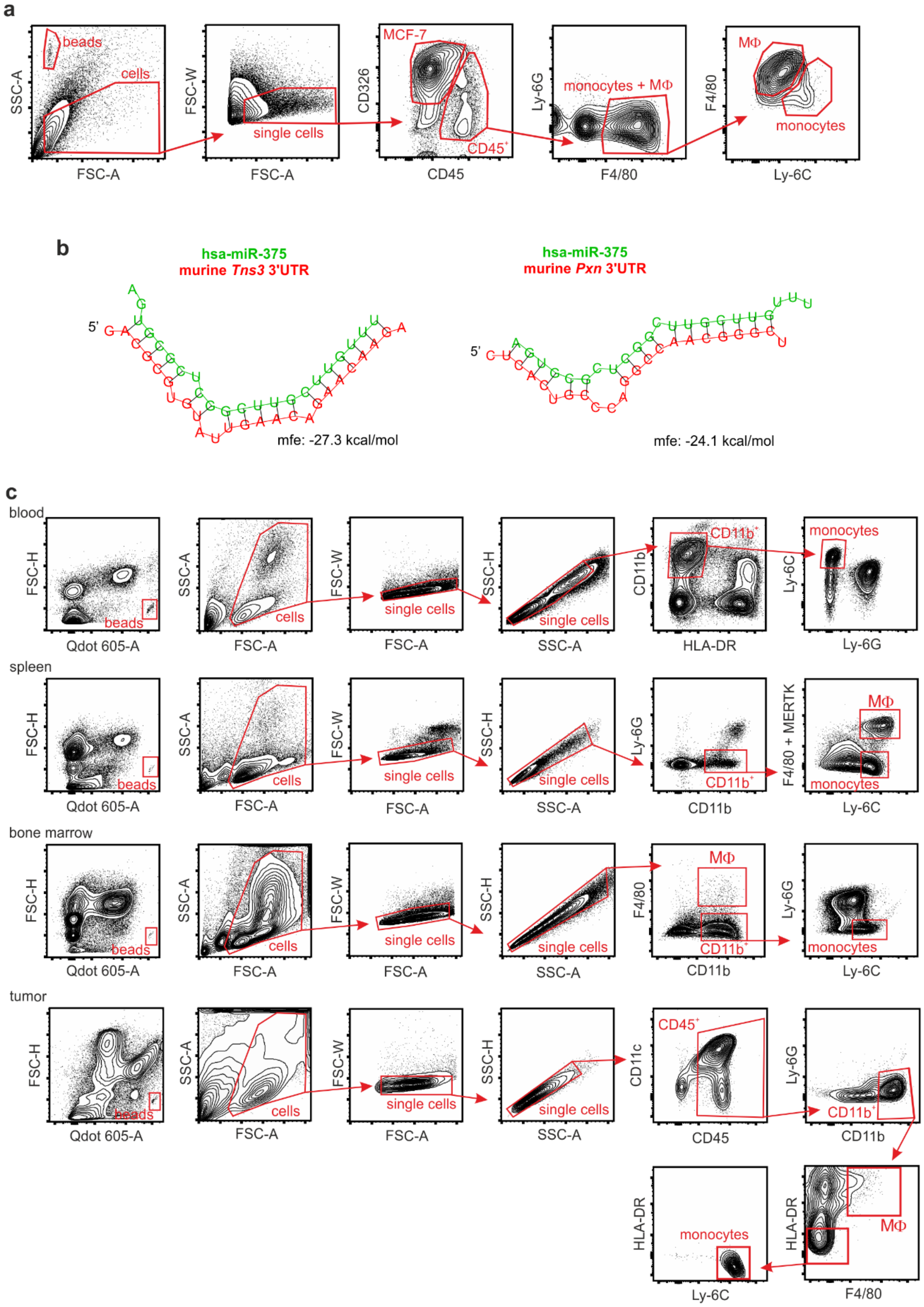

Supplementary Fig. 11 - a Gating strategy to discriminate MCF-7 cells and tumor infiltrating murine monocytes and MΦ by flow cytometry. Single-cell populations were gated for CD326^{+}

MCF-7 cells and CD45$^+$ immune cells. F4/80$^+$ cells were further divided into F4/80^{hi} MΦ and F4/80^{low} and Ly-6C^{hi} monocytes. **b** Analysis of 3'UTR of murine *Tns3* and *Pxn* for human miR-375 binding with the RNAhybrid tool (PMID: 15383676). Negative minimum free energy (mfe) signifies physiological binding. **c** 50000 E0771 control (control empty vector) or decoy (miR-375 decoy) cells were injected in mammary gland 3 and 8 of 8 weeks old female C57BL/6 mice. After 14 days blood, bone marrow, spleen and tumors were harvested for polychromatic flow cytometry and cell sorting. Gating strategy to separate tumor infiltrating monocytes and MΦ as well as monocytes and MΦ from blood, spleen and bone marrow by flow cytometry.

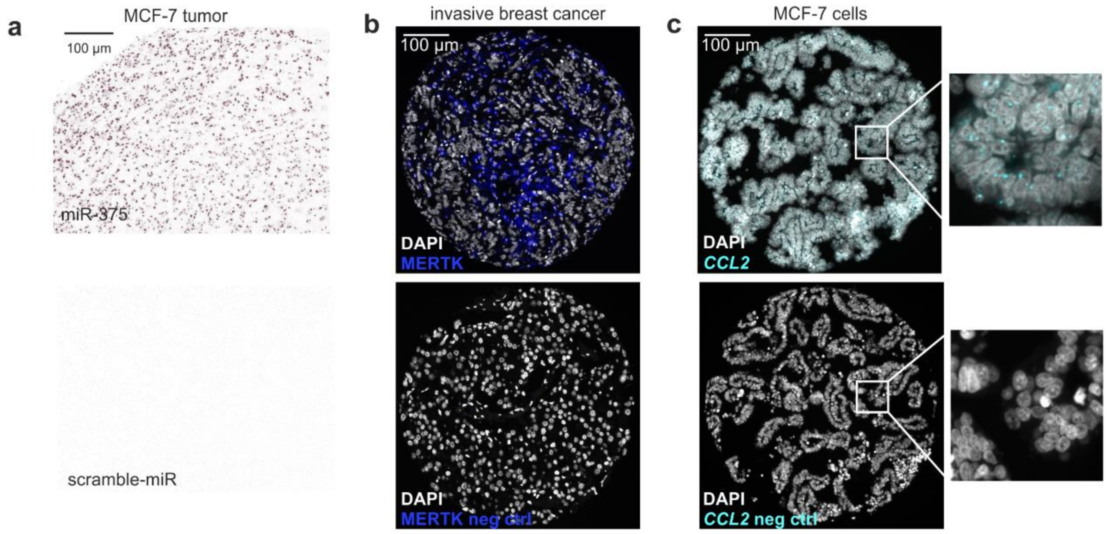

Supplementary Fig. 12 - a Sections of MCF-7 tumor from NMRI-*Foxn1^{nu}* mice (Fig. 8b) were used to establish miR-375 *in situ* hybridization. Sections were hybridized with 40 nM of double DIG labeled miRCURY LNA™ miRNA Detection Probe for hsa-miR-375 (top) or scramble-miR (bottom) for 1 h at 55°C. Representative images of positive miR-375 signals as well as negative controls (scramble-miR) are shown. **b** Human mammary carcinoma tissue microarrays slides were stained with DAPI (nuclei), anti-MERTK antibody (top) or control (bottom; neg ctrl) using Opal staining system. **c** MCF-7 cells from human mammary carcinoma tissue microarrays slides were probed with HS-CCL2 (top) or negative control (bottom; neg ctrl) by RNAscope® technique. Representative section with arrowheads in magnification showing positive *CCL2* (top) and negative (bottom) signals. Nuclei were stained with DAPI (white).

Supplementary Table 1 - Primers used in qPCR

**gene name**	**sense (5'- 3')**	**anti-sense (5'- 3')**
human *TBP*	GGGCCGCCGGCTGTTTAACT	AGCCCTGAGCGTAAGGTGGCA
human *IL1B*	TCTTTAACGCAGGACAG	TTCGACACATGGGATAACGA
human *IL12*	ACCCTGACCATCCAAGTCAAA	TTGGCCTCGCATCTTAGAAAG
human *CLEC7A*	CCGGTAAGTACCTAGCCCACA	GCTCCTGAGATGACTGTCTGT
human *RNA18S*	GTAACCCGTTGAACCCCATT	CCATCCAATCGGTAGTAGCG
human *PXN*	CTGCTGGAACTGAACGCTGTA	GGGGCTGTTAGTCTCTGGGA
human *TNS3*	ACCTCACTTACATCACGGAGC	GTAGGTTGTGCAGGTAGGACT
human *CXCL10*	GTGGCATTCAAGGAGTACCTC	TGATGGCCTTCGATTCTGGATT
human *CCL2*	CAGCCAGATGCAATCAATGCC	TGGAATCCTGAACCCACTTCT
human *CCL5*	CCAGCAGTCGTCTTTGTCAC	CTCTGGGTTGGCACACACTT
human *DICER*	TGCTATGTCGCCTTGAATGTT	AATTTCTCGATAGGGGTGGTCTA
human *JAK2*	TACCTCTTTGCTCAGTGGCG	AACACTGCCATCCCAAGACA
human *PDK1*	GAGAGCCACTATGGAACACCA	GGAGGTCTCAACACGAGGT
human *SPAG9*	CAAGCACTCCCACCAAAGG	CCCGACCCATTCCTAGTAAATCT
human *pre-miR 375*	CCGCGACGAGCCCCT	CCGAACGAACAAAACGCTCA
mouse *Rps27a*	GACCCTTACGGGGAAAACCAT	AGACAAAGTCCGGCCATCTTC
mouse *Pxn*	CAGTGGCAGCCTAGTGGTTC	GGGAAGCTGTAGACGTGCTC
mouse *Tns3*	CAGGGGTGGTAAAGGACGC	GGAGGGCTCCATTAAAGCTGAA
mouse *Rnu6*	CTTCGGCAGCACATATACTAAAAT	

Supplementary Table 2 - Cell lines and culture media

Cell line (supplier)	Disease/cell type	Medium
HCC-1937 (ATCC)	human lymphoblast, epithelial, breast cancer TNM stage IIB, grade 3, primary ductal carcinoma	DMEM +10% FCS + 1% sodium pyruvate + 100 U/mL penicillin + 100 µg/mL streptomycin
SKBR3 (DSMZ)	human mammary gland/breast; derived from metastatic site: pleural effusion; adenocarcinoma	
EFM-192A (DSMZ)	human breast adenocarcinoma; pleural effusion	RPMI 1640 + 20% FCS +100 U/mL penicillin +100 µg/mL streptomycin
MDA-MB-231 (ATCC)	human mammary gland/breast cancer; derived from metastatic site: pleural effusion	DMEM + 10% FCS + 100 U/mL penicillin + 100 µg/mL streptomycin
MDA-MB-468 (ATCC)	human mammary gland/breast cancer; derived from metastatic site: pleural effusion	
A375 (ATCC)	human malignant melanoma	
A549 (ATCC)	human lung carcinoma	
T98G (ATCC)	human brain glioblastoma	
HEK293T (ATCC)	human embryonic kidney	
T47D (ATCC)	human mammary gland, cancer; derived from metastatic site: pleural effusion	RPMI 1640 + 10% FCS + 100 U/mL penicillin +100 µg/mL streptomycin
Jurkat (ATCC)	human T lymphocyte	
MCF-10A (ATCC)	human mammary gland; breast; fibrocystic disease	DMEM + 10% FCS + 15 mM HEPES + 1% MEM + 1% P/S + 100 ng/mL hydrocortisone + 100 ng/mL insulin + 100 ng/mL EGF + 100 ng/mL cholera toxin
HMEC (ATCC)	human primary mammary epithelial cells; normal	Mammary epithelial cell basal medium (ATCC) Mammary + epithelial cell growth kit (ATCC)
143B (ATCC)	human bone osteosarcoma	MEM Earle's BSS + 0.015 mg/mL 5-bromo-2'-deoxyuridine (Sigma-Aldrich) + 100 U/mL penicillin + 100 µg/mL streptomycin
E0771 (CH3 BioSystems)	murine malignant neoplasm of the mammary gland	RPMI 1640 +10% FCS + 10 mmol/L HEPES (Sigma-Aldrich) + 100 U/mL penicillin + 100 µg/mL streptomycin
