# Supplementary material for: Apoptotic tumor cell-derived microRNA-375 uses CD36 to alter the tumor-associated macrophage phenotype
Source: Nat Commun. 2019 Mar 8;10:1135. doi: 10.1038/s41467-019-08989-2 (PMC6408494; doi:10.1038/s41467-019-08989-2)
Supplement: Supplementary file 3 — Description of Additional Supplementary Files [file 41467_2019_8989_MOESM3_ESM.docx]

**Description of Additional Supplementary Files**

File Name: Supplementary Movie 1

Description: 3D volume rendering of control MCF-7 spheroids infiltrated with eFluor670 labeled CD14+ monocytes. Area occupied by infiltrating monocytes is denoted by white, whereas total spheroid area is in blue.

File Name: Supplementary Movie 2

Description: 3D volume rendering of miR-375 decoy MCF-7 spheroids infiltrated with eFluor670 labeled CD14+ monocytes. Area occupied by infiltrating monocytes is denoted by white, whereas total spheroid area is in blue.

File Name: Supplementary Data 1

Description: Differentially expressed miRNA genes in miRseq of primary human MΦ control, cocultured with MCF-7 cells, and treated with RvD1. Normalized mature miRNA expression table using multi mapper strategy by number of reads per million (RPM).

File Name: Supplementary Data 2

Description: Potential direct miR-375 targets identified in primary human MΦ by Ago-RIP-Seq. Normalized mRNA expression table of linear number of reads per kilobase of transcripts per million mapped reads (RPKM).
