# Supplementary material for: Apoptotic tumor cell-derived microRNA-375 uses CD36 to alter the tumor-associated macrophage phenotype
Source: Nat Commun. 2019 Mar 8;10:1135. doi: 10.1038/s41467-019-08989-2 (PMC6408494; doi:10.1038/s41467-019-08989-2)
Supplement: Supplementary file 8 — Reporting Summary [file 41467_2019_8989_MOESM8_ESM.pdf]

nature research

Corresponding author(s): Shahzad N. Syed and Bernhard Brüne

Last updated by author(s): Jan 23, 2019

Reporting Summary

Nature Research wishes to improve the reproducibility of the work that we publish. This form provides structure for consistency and transparency in reporting. For further information on Nature Research policies, see Authors & Referees and the Editorial Policy Checklist.

Statistics

For all statistical analyses, confirm that the following items are present in the figure legend, table legend, main text, or Methods section.

n/a	Confirmed	
☐	☒	The exact sample size (*n*) for each experimental group/condition, given as a discrete number and unit of measurement
☐	☒	A statement on whether measurements were taken from distinct samples or whether the same sample was measured repeatedly
☐	☒	The statistical test(s) used AND whether they are one- or two-sided<br>*Only common tests should be described solely by name; describe more complex techniques in the Methods section.*
☒	☐	A description of all covariates tested
☒	☐	A description of any assumptions or corrections, such as tests of normality and adjustment for multiple comparisons
☐	☒	A full description of the statistical parameters including central tendency (e.g. means) or other basic estimates (e.g. regression coefficient) AND variation (e.g. standard deviation) or associated estimates of uncertainty (e.g. confidence intervals)
☐	☒	For null hypothesis testing, the test statistic (e.g. *F*, *t*, *r*) with confidence intervals, effect sizes, degrees of freedom and *P* value noted<br>*Give P values as exact values whenever suitable.*
☒	☐	For Bayesian analysis, information on the choice of priors and Markov chain Monte Carlo settings
☒	☐	For hierarchical and complex designs, identification of the appropriate level for tests and full reporting of outcomes
☒	☐	Estimates of effect sizes (e.g. Cohen's *d*, Pearson's *r*), indicating how they were calculated

Our web collection on statistics for biologists contains articles on many of the points above.

Software and code

Policy information about availability of computer code

Data collection	No software was used other than that listed in the Methods part of the manuscript (e.g., NextSeq 500 for genome sequencing) or standard software provided by manufacturers of data collection instruments (Illumina sequencers, Becten-Dickenson FACS, Agilient Bioanlyzer, BioRad qPCR, Zeiss microscope, Li-Cor Odyssey WB, Mithras LB 940 luminometer, Ultramicroscope II LSM etc.). The respective analysis tools provided by the instrument vendors are listed in the methods.
Data analysis	Illumina Experiment Manager 1.15.1; fastqc 0.11.8; bowtie 1.0.0; Trimmomatic; samtools; DESeq2 1.18.1; R package 3.4.2; DNASTAR SeqMan NGen 12.3.1.4; ArrayStar 12.3.1; Genome sequence analysis, BD FACSDiva 8.0.1; FACS sorting, CFX Manager 3.2; qPCR data anlysis, ImageJ2 1.52; Scratch assay, Image Lab 4.1; WB analysis, Vectra3, inForm 2.0; IHC and RNAscope, Flowjo v10; Flow cytometry, MikroWin 2010; luciferase assay, FCAP 1.0.1; CBA, ImSpectorPro 5.0; Imaris 7.6; light sheet microscopy, GraphPad Prism 8.0 for plotting bar graphs; CorelDraw X8 for vector images and clip arts.

For manuscripts utilizing custom algorithms or software that are central to the research but not yet described in published literature, software must be made available to editors/reviewers. We strongly encourage code deposition in a community repository (e.g. GitHub). See the Nature Research guidelines for submitting code & software for further information.

Data

Policy information about availability of data

All manuscripts must include a data availability statement. This statement should provide the following information, where applicable:

- Accession codes, unique identifiers, or web links for publicly available datasets
- A list of figures that have associated raw data
- A description of any restrictions on data availability

All sequencing data supporting the findings of this study have been uploaded to the the ArrayExpress database at EMBL-EBI under the accession numbers E-MTAB-6885 [https://www.ebi.ac.uk/arrayexpress/experiments/E-MTAB-6885/] and E-MTAB-6943 [https://www.ebi.ac.uk/arrayexpress/experiments/E-MTAB-6943] respectively. All relevant data are within the paper and its supplementary information files. Any further data not included in the manuscript is available from the

October 2018

corresponding author on reasonable request.

Field-specific reporting

Please select the one below that is the best fit for your research. If you are not sure, read the appropriate sections before making your selection.

☒ Life sciences ☐ Behavioural & social sciences ☐ Ecological, evolutionary & environmental sciences

For a reference copy of the document with all sections, see nature.com/documents/nr-reporting-summary-flat.pdf

Life sciences study design

All studies must disclose on these points even when the disclosure is negative.

Sample size	Sample-size calculations were not required for all experiments of this study. Experiments were independently repeated at least twice as indicated, and mean and the standard error from the mean were calculated.
Data exclusions	No data have been excluded from the analysis.
Replication	All attempts at replication were successful. Experiments were carried out in biological and/or technical replicates as indicated in the results part (text, figure legends, tables).
Randomization	Randomization was not relevant in this study as there was no comparison of cohorts
Blinding	Investigators were blinded to group allocation for scratch assays, tumor growth scoring, light sheet microscopy, in situ hybridization, multiplex immunohistochemistry and cytometric bead array. All other experiments were performed in non-blinded manner.

Reporting for specific materials, systems and methods

We require information from authors about some types of materials, experimental systems and methods used in many studies. Here, indicate whether each material, system or method listed is relevant to your study. If you are not sure if a list item applies to your research, read the appropriate section before selecting a response.

Materials & experimental systems

n/a	Involved in the study	
☐	☒	Antibodies
☐	☒	Eukaryotic cell lines
☒	☐	Palaeontology
☐	☒	Animals and other organisms
☒	☐	Human research participants
☐	☒	Clinical data

Methods

n/a	Involved in the study	
☒	☐	ChIP-seq
☐	☒	Flow cytometry
☒	☐	MRI-based neuroimaging

Antibodies

Antibodies used	CD36 blocking antibody (ImmunoTools #21270361), IgG control (ImmunoTools #21915011), CD14 microbeads (Miltenyi Biotec #130-050-201), anti-PXN (Abcam #ab32084), anti-TNS3 (ThermoFisher Scientific #PA5-63112) and anti-nucleolin (Santa Cruz biotechnology #sc-8031), anti-MERTK (Abcam #52968), CD80-APC (BioLegend, #305220),), CD86-FITC (BD Bioscience #555657), CD206-PE-Cy5 (BioLegend #321108), CD163-PE (BD Bioscience #558018), HLA-DR-PE-Cy7 (BD Bioscience #560651), CD45-PE (BioLegend #368509), CD14-APC-H7 (BD Bioscience #560180), CD11c-V450 (BD Bioscience #560370), CD11b-eFluor605 (BioLegend #101257), F4/80-Pe-Cy7 (BioLegend #123114), Ly-6G-APC-Cy7 (BioLgened #127624), CD326-PE (BioLegend #324205), CD11c-BV711 (BD Bioscience #363048), Ly-6c-PerCP-PE-Cy5.5 (BD Bioscience #560525), CD45-VioBlue (Miltenyi Biotec #130102430), HLA-DR-APC (Miltenyi Biotec #130102139), Anti-pan-Ago IgG 2A8 (Merck Millipore #MABE56), IgG1 isotype control (ImmunoTools #21275511)
Validation	Validation statements for all the antibodies used in the study are available at the websites of the respective commercial providers. For flow cytometry, FMO control were used for compensation.

Eukaryotic cell lines

Policy information about cell lines

Cell line source(s)	HCC-1937 (ATCC), SKBR3 (DSMZ), EFM-192A (DSMZ), MDA-MB-231 (ATCC), MDA-MB-468 (ATCC), A375 (ATCC), A549 (ATCC), T98G (ATCC), HEK293T (ATCC), T47D (ATCC), Jurkat (ATCC), MCF-7 (ATCC), MCF-10A (ATCC), HMEC (ATCC), 143B (ATCC), E0771 (CH3 BioSystems).

Authentication	Cell lines were obtained from original sources and were not further authenticated.
Mycoplasma contamination	All (human and mouse) cell lines were routinely tested for potential contaminations with mycoplasma using commercial PCR-based kits Venor® GeM (Minerva Biolabs, #11-1100). All tests were negative.
Commonly misidentified lines (See ICLAC register)	does not apply

Animals and other organisms

Policy information about studies involving animals; ARRIVE guidelines recommended for reporting animal research

Laboratory animals	8-12 weeks old female NMRI-Foxn1nu mice and 8 weeks old female C57BL/6 mice. Housing and organ isolation were done in accordance with European guidelines and the German Animal Protection Law.
Wild animals	does not apply
Field-collected samples	does not apply
Ethics oversight	The Hessian animal care and use committee (approval no. FU/1152) and University Animal Welfare Office according to Animal Protection Act (§10 i.V.m §5 Animal Protection Laboratory Animal Ordinance).

Note that full information on the approval of the study protocol must also be provided in the manuscript.

Clinical data

Policy information about clinical studies

All manuscripts should comply with the ICMJE guidelines for publication of clinical research and a completed CONSORT checklist must be included with all submissions.

Clinical trial registration	does not apply
Study protocol	does not apply
Data collection	does not apply
Outcomes	does not apply

Flow Cytometry

Plots

Confirm that:

- [ ] The axis labels state the marker and fluorochrome used (e.g. CD4-FITC).
- [x] The axis scales are clearly visible. Include numbers along axes only for bottom left plot of group (a 'group' is an analysis of identical markers).
- [x] All plots are contour plots with outliers or pseudocolor plots.
- [x] A numerical value for number of cells or percentage (with statistics) is provided.

Methodology

Sample preparation	To analyze macrophage phenotypes, cells were washed with PBS and pelleted at 500 x g and 4°C for 5 min. For analysis of infiltrated spheroids, cocultures were harvested and washed with PBS to remove non-infiltrating monocytes, followed by treatment with accutase (PAA Laboratories, Cölbe, Germany) for 30 min at 37°C to obtain single-cell suspensions. Cells were washed with PBS and pelleted at 500 x g and 4°C for 5 min. For FACS analysis and FACS sorting of MCF-7 tumors from xenograft transplantation experiments, single-cell suspensions were created using the human tumor dissociation kit and the GentleMACS isolator (Miltenyi Biotec, Gladbach, Germany). Single-cell suspension from murine E0771 tumors were prepared using the murine tumor dissociation kit (Miltenyi Biotec).
Instrument	LSRII/Fortessa (BD Biosciences), a FACSAria III (BD Biosciences)
Software	BD FACS Diva (BD Biosciences) for acquisition and FlowJo software V10 (Treestar) for analysis
Cell population abundance	To measure monocyte/macrophage abundance in MCF-7 tumor spheroids, single-cell populations were gated for GFP-expressing MCF-7 control or decoy cells and CD14+ cells, which were further gated for CD11c+ macrophages. As an internal counting standard for later analysis, flow-count fluorospheres (beads) were gated and used for data normalization. For other tumor spheroids, single-cell suspensions were gated for CD14+/CD45+ immune cells followed by gating for CD11c+/CD14+ macrophage. To check the abundance of monocytes/macrophages in xenograft tumor model, Single-cell populations were gated for CD326+ MCF-7 cells and CD45+ immune cells. F4/80+ cells were further divided into F4/80hi macrophages and F4/80low and Ly-6Chi monocytes.

Gating strategy

Main cell population was gated in FSC-A/SSC-A excluding debris and douplets. For infiltration of CD14+ cells in tumor spheroids, gating strategy is described in Supplementary Fig. 9b and 10c. For monocyte and macrophage infiltration into xenograft MCF-7 tumor, gating strategy is described in Supplementary Fig. 11a. For orthotopically transplanted model, gating strategy is described in Supplementary Fig. 11c.

☒ Tick this box to confirm that a figure exemplifying the gating strategy is provided in the Supplementary Information.
